# Expanding a database-derived biomedical knowledge graph via multi-relation extraction from biomedical abstracts

**DOI:** 10.1186/s13040-022-00311-z

**Published:** 2022-10-18

**Authors:** David N. Nicholson, Daniel S. Himmelstein, Casey S. Greene

**Affiliations:** 1 Department of Systems Pharmacology and Translational Therapeutics, University of Pennsylvania, Philadelphia, PA USA; 2 Department of Biomedical Informatics, University of Colorado School of Medicine and Center for Health Artificial Intellegence (CHAI), University of Colorado School of Medicine, Aurora, USA

## Abstract

**Background:**

Knowledge graphs support biomedical research efforts by providing contextual information for biomedical entities, constructing networks, and supporting the interpretation of high-throughput analyses. These databases are populated via manual curation, which is challenging to scale with an exponentially rising publication rate. Data programming is a paradigm that circumvents this arduous manual process by combining databases with simple rules and heuristics written as label functions, which are programs designed to annotate textual data automatically. Unfortunately, writing a useful label function requires substantial error analysis and is a nontrivial task that takes multiple days per function. This bottleneck makes populating a knowledge graph with multiple nodes and edge types practically infeasible. Thus, we sought to accelerate the label function creation process by evaluating how label functions can be re-used across multiple edge types.

**Results:**

We obtained entity-tagged abstracts and subsetted these entities to only contain compounds, genes, and disease mentions. We extracted sentences containing co-mentions of certain biomedical entities contained in a previously described knowledge graph, Hetionet v1. We trained a baseline model that used database-only label functions and then used a sampling approach to measure how well adding edge-specific or edge-mismatch label function combinations improved over our baseline. Next, we trained a discriminator model to detect sentences that indicated a biomedical relationship and then estimated the number of edge types that could be recalled and added to Hetionet v1. We found that adding edge-mismatch label functions rarely improved relationship extraction, while control edge-specific label functions did. There were two exceptions to this trend, Compound-binds-Gene and Gene-interacts-Gene, which both indicated physical relationships and showed signs of transferability. Across the scenarios tested, discriminative model performance strongly depends on generated annotations. Using the best discriminative model for each edge type, we recalled close to 30% of established edges within Hetionet v1.

**Conclusions:**

Our results show that this framework can incorporate novel edges into our source knowledge graph. However, results with label function transfer were mixed. Only label functions describing very similar edge types supported improved performance when transferred. We expect that the continued development of this strategy may provide essential building blocks to populating biomedical knowledge graphs with discoveries, ensuring that these resources include cutting-edge results.

**Supplementary Information:**

The online version contains supplementary material available at 10.1186/s13040-022-00311-z.

## Introduction

Knowledge bases are essential resources that hold complex structured and unstructured information. These resources have been used to construct networks for drug repurposing discovery [1–3] or as a source of training labels for text mining systems [4–6]. Populating knowledge bases often requires highly trained scientists to read biomedical literature and summarize the results through manual curation [7]. In 2007, researchers estimated that filling a knowledge base via manual curation would require approximately 8.4 Years to complete [8]. As the rate of publications increases exponentially [9], using only manual curation to populate a knowledge base has become nearly impractical.

Relationship extraction is one of several solutions to the challenge posed by an exponentially growing body of literature [7]. This process creates an expert system to automatically scan, detect, and extract relationships from textual sources. These expert systems fall into three types: unsupervised, rule-based, and supervised systems.

Unsupervised systems extract relationships without the need for annotated text. These approaches utilize linguistic patterns such as the frequency of two entities appearing in a sentence together more often than chance, commonly referred to as co-occurrence [10–18]. For example, a possible system would say gene X is associated with disease Y because gene X and disease Y appear together more often than chance [10]. Besides frequency, other systems can utilize grammatical structure to identify relationships [19]. This information is modeled in the form of a tree data structure, termed a dependency tree. Dependency trees depict words as nodes, and edges represent a word’s grammatical relationship with one another. Through clustering on these generated trees, one can identify patterns that indicate a biomedical relationship [19]. Unsupervised systems are desirable since they do not require well-annotated training data; however, precision may be limited compared to supervised machine learning systems.

Rule-based systems rely heavily on expert knowledge to perform relationship extraction. These systems use linguistic rules and heuristics to identify critical sentences or phrases that suggest the presence of a biomedical relationship [20–25]. For example, a hypothetical extractor focused on protein phosphorylation events would identify sentences containing the phrase “gene X phosphorylates gene Y” [20]. These approaches provide exact results, but the quantity of positive results remains modest as sentences consistently change in form and structure. For this project, we constructed our label functions without the aid of these works; however, the approaches mentioned in this section provide substantial inspiration for novel label functions in future endeavors.

Supervised systems depend on machine learning classifiers to predict the existence of a relationship using biomedical text as input. These classifiers can range from linear methods such as support vector machines [26, 27] to deep learning [28–33], which all require access to well-annotated datasets. Typically, these datasets are usually constructed via manual curation by individual scientists [34–38] or through community-based efforts [39–41]. Often, these datasets are well annotated but are modest in size, making model training hard as these algorithms become increasingly complex.

Distant supervision is a paradigm that quickly sidesteps manual curation to generate large training datasets. This technique assumes that positive examples have been previously established in selected databases, implying that the corresponding sentences or data points are also positive [4]. The central problem with this technique is that generated labels are often of low quality, resulting in many false positives [42]. Despite this caveat there have been notable effort using this technique [43–45].

Data programming is one proposed solution to amend the false positive problem in distant supervision. This strategy combines labels obtained from distant supervision with simple rules and heuristics written as small programs called label functions [46]. These outputs are consolidated via a noise-aware model to produce training labels for large datasets. Using this paradigm can dramatically reduce the time required to obtain sufficient training data; however, writing a helpful label function requires substantial time and error analysis. This dependency makes constructing a knowledge base with a myriad of heterogenous relationships nearly impossible as tens or hundreds of label functions are necessary per relationship type.

This paper seeks to accelerate the label function creation process by measuring how label functions can be reused across different relationship types. We hypothesized that sentences describing one relationship type might share linguistic features such as keywords or sentence structure with sentences describing other relationship types. If this hypothesis were to, one could drastically reduce the time needed to build a relation extractor system and swiftly populate large databases like Hetionet v1. We conducted a series of experiments to estimate how label function reuse enhances performance over distant supervision alone. As biomedical data comes in various forms (e.g. publications, electronic health records, images, genomic sequences, etc.), we chose to subset this space to only include open-access biomedical publications available on pubmed. We focused on relationships that indicated similar types of physical interactions (i.e., Gene-binds-Gene and Compound-binds-Gene) and two more distinct types (i.e., Disease-associates-Gene and Compound-treats-Disease).

## Methods and materials

### Hetionet

**Fig. 1:**
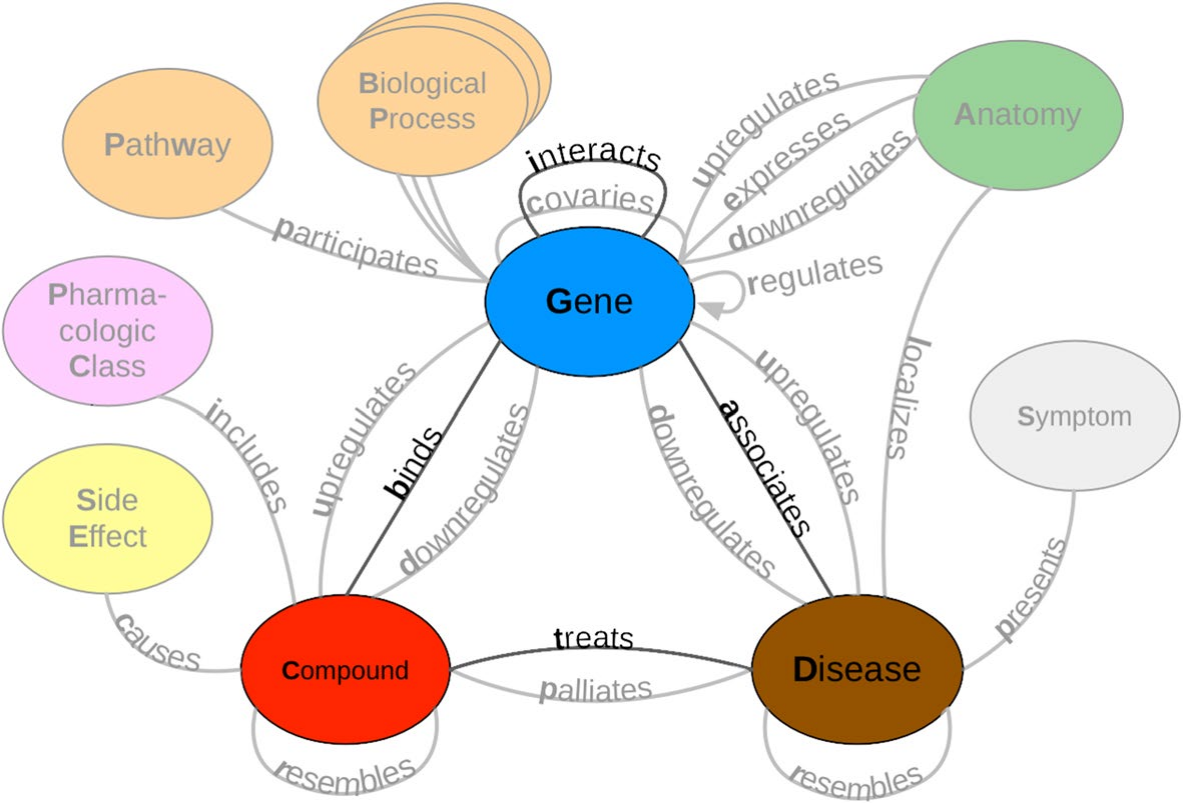
A metagraph (schema) of Hetionet v1 where biomedical entities are represented as nodes and the relationships between them are represented as edges. We examined performance on the highlighted subgraph; however, the long-term vision is to capture edges for the entire graph

Hetionet v1 [3] is a heterogeneous network that contains pharmacological and biological information. This network depicts information in the form of nodes and edges of different types. Nodes in this network represent biological and pharmacological entities, while edges represent relationships between entities. Hetionet v1 contains 47,031 nodes with 11 different data types and 2,250,197 edges that represent 24 different relationship types (Fig. 1). Edges in Hetionet v1 were obtained from open databases, such as the GWAS Catalog [47], Human Interaction database [48] and DrugBank [49]. For this project, we analyzed performance over a subset of the Hetionet v1 edge types: disease associates with a gene (DaG), compound binds to a gene (CbG), compound treating a disease (CtD), and gene interacts with gene (GiG) (bolded in Fig. 1).

### Dataset

We used PubTator Central [50] as input to our analysis. PubTator Central provides MEDLINE abstracts that have been annotated with well-established entity recognition tools including Tagger One [51] for disease, chemical and cell line entities, tmVar [52] for genetic variation tagging, GNormPlus [53] for gene entities and SR4GN [54] for species entities. We downloaded PubTator Central on March 1, 2020, at which point it contained approximately 30,000,000 documents. After downloading, we filtered out annotated entities that were not contained in Hetionet v1. We extracted sentences with two or more annotations and termed these sentences as candidate sentences. We used the Spacy’s English natural language processing (NLP) pipeline (en_core_web_sm) [55] to generate dependency trees and parts of speech tags for every extracted candidate sentence. Each candidate sentence was stratified by their corresponding abstract ID to produce a training set, tuning set, and a testing set. We used random assortment to assign dataset labels to each abstract. Every abstract had a 70% chance of being labeled training, 20% chance of being labeled tuning, and 10% chance of being labeled testing. Despite the power of data programming, all text mining systems need to have ground truth labels to be well-calibrated. We hand-labeled five hundred to a thousand candidate sentences of each edge type to obtain a ground truth set (Table 1).

**Table 1:** Statistics of Candidate Sentences. We sorted each abstract into a training, tuning and testing set. Numbers in parentheses show the number of positives and negatives that resulted from the hand-labeling process

Relationship	Train	Tune	Test
Disease-associates-Gene (DaG)	2.49 M	696 K (397 + , 603-)	348 K (351 + , 649-)
Compound-binds-Gene (CbG)	2.4 M	684 K (37 + , 463-)	341 k (31 + , 469-)
Compound-treats-Disease (CtD)	1.5 M	441 K (96 + , 404-)	223 K (112 + , 388-)
Gene-interacts-Gene (GiG)	11.2 M	2.19 M (60 + , 440-)	1.62 M (76 + , 424-)

### Label functions for annotating sentences

 The challenge of having too few ground truth annotations is familiar to many biomedical applications that use natural language processing, even when unannotated text is abundant. Data programming circumvents this issue by quickly annotating large datasets using multiple noisy signals emitted by label functions [46]. We chose to use data programming for this project as it allows us to provide generalizable rules that can be reused in future text mining systems. Label functions are simple pythonic functions that emit: a positive label (1), a negative label (0), or abstain from emitting a label (-1). These functions can use different approaches or techniques to emit a label; however, these functions can be grouped into simple categories discussed below. Once constructed, these functions are combined using a generative model to output a single annotation. This single annotation is a consensus probability score bounded between 0 (low chance of mentioning a relationship) and 1 (high chance of mentioning a relationship). We used these annotations to train a discriminative model for the final classification step.

#### Label function categories

Label functions can be constructed in various ways; however, they also share similar characteristics. We grouped functions into databases and text patterns. The majority of our label functions fall into the text pattern category (Table 2). Further, we described each label function category and provided an example that refers to the following candidate sentence: “PTK6 may be a novel therapeutic target for pancreatic cancer”.

**Table 2:** The distribution of each label function per relationship

Relationship	Databases (DB)	Text Patterns (TP)
DaG	7	30
CtD	3	22
CbG	9	20
GiG	9	28

#### Databases

These label functions incorporate existing databases to generate a signal, as seen in distant supervision [4]. These functions detect if a candidate sentence’s co-mention pair is present in a given database. Our label function emits a positive label if the pair is present and abstains otherwise. If the pair is not present in any existing database, a separate label function emits a negative label. We used a separate label function to prevent a label imbalance problem, which can occur when a single function labels every possible sentence despite being correct or not. If this problem isn’t handled correctly, the generative model could become biased and only emit one prediction (solely positive or solely negative) for every sentence.

$$\Lambda_{DB}\left(D,G\right)=\left\{\begin{array}{ll}1&\left(D,\;G\right)\;\in DB\\0&otherwise\end{array}\right.$$

$$\Lambda_{\neg DB}\left(D,G\right)=\left\{\begin{array}{ll}-1 &\left(D,G\right)\;\not\in DB\\0&otherwise\end{array}\right.$$

#### Text patterns

These label functions are designed to use keywords or sentence context to generate a signal. For example, a label function could focus on the number of words between two mentions and emit a label if two mentions are too close. Alternatively, a label function could focus on the parts of speech contained within a sentence and ensures a verb is present. Besides parts of speech, a label function could exploit dependency parse trees to emit a label. These trees are akin to the tree data structure where words are nodes and edges are how each word modifies each other. Label functions that use these parse trees will test if the generated tree matches a pattern and emits a positive label if true. For our analysis, we used previously identified patterns designed for biomedical text to generate our label functions [19].

$$\Lambda_{TP}\left(D,G\right)=\left\{\begin{array}{ll}1&"target"\in\textit{Candidate Sentence}\\-1 & \textit{otherwise}\end{array}\right.$$

$$\Lambda_{TP}\left(D,G\right)=\left\{\begin{array}{ll} 0&"VB"\;\not\in\;pos\_tags\left(Candidate\;Sentence\right)\\-1&otherwise\end{array}\right.$$

$$\Lambda_{TP}\left(D,G\right) = \left\{ \begin{array}{ll} 1 & dep\left(Candidate\;Sentence\right)\;\in\;Cluster\;Theme\\ -1 & otherwise\end{array}\right.$$

Each text pattern label function was constructed via manual examination of sentences within the training set. For example, using the candidate sentence above, one would identify the phrase “novel therapeutic target” and incorporate this phrase into a global list that a label function would use to check if present in a sentence. After initial construction, we tested and augmented the label function using sentences in the tune set. We repeated this process for every label function in our repertoire.

### Training models

#### Generative model

The generative model is a core part of this automatic annotation framework. It integrates multiple signals emitted by label functions to assign each candidate sentence the most appropriate training class. This model takes as input a label function output in the form of a matrix where rows represent candidate sentences, and columns represent each label function (${\Lambda }^{nxm}$). Once constructed, this model treats the true training class ($Y$) as a latent variable and assumes that each label function is independent of one another. Under these two assumptions, the model finds the optimal parameters by minimizing a loglikelihood function marginalized over the latent training class.

$$\widehat\theta=argmin_\theta\sum\limits_Y-log\left(P_\theta\left(\Lambda,Y\right)\right)$$

Following optimization, the model emits a probability estimate that each sentence belongs to the positive training class. At this step, each probability estimate can be discretized via a chosen threshold into a positive or negative class. This model uses the following parameters to generate training estimates: weight for the l2 loss, a learning rate, and the number of epochs. We fixed the learning rate to be 1e-3 as we found that higher weights produced NaN results. We also fixed the number of epochs to 250 and performed a grid search of five evenly spaced numbers between 0.01 and 5 for the l2 loss parameter. Following the training phase, we used a threshold of 0.5 for discretizing training classes’ probability estimates within our analysis. For more information on how the likelihood function is constructed and minimized, refer to [56].

#### Discriminative model

The discriminative model is the final step in this framework. This model uses training labels generated from the generative model combined with sentence features to classify the presence of a biomedical relationship. Typically, the discriminative model is a neural network. In the context of text mining, these networks take the form of transformer models [32], which have achieved high-performing results. Their past performance lead us to choose BioBERT [31] as our discriminative model. BioBERT [31] is a BERT [57] model that was trained on all papers and abstracts within Pubmed Central [58]. BioBERT provides its own set of word embeddings, dense vectors representing words that models such as neural networks can use to construct sentence features. We downloaded a pre-trained version of this model using huggingface’s transformer python package [59] and fine-tuned it using our generated training labels. Our fine-tuning approach involved freezing all downstream layers except for the classification head of this model. Next, we trained this model for 10 epochs using the Adam optimizer [60] with huggingface’s default parameter settings and a learning rate of 0.001.

### Experimental design

Reusing label functions across edge types would substantially reduce the number of label functions required to extract multiple relationships from biomedical literature. We first established a baseline by training a generative model using only distant supervision label functions designed for the target edge type. Then we compared the baseline model with models that incorporated a set number of text pattern label functions. Using a sampling with replacement approach, we sampled these text pattern label functions from three different groups: within edge types, across edge types, and from a pool of all label functions. We compared within-edge-type performance to across-edge-type and all-edge-type performance. We sampled a fixed number of label functions for each edge type consisting of five evenly spaced numbers between one and the total number of possible label functions. We repeated this sampling process 50 times for each point. Furthermore, we also trained the discriminative model using annotations from the generative model trained on edge-specific label functions at each point. We report the performance of both models in terms of the area under the receiver operating characteristic curve (AUROC) and the area under the precision-recall curve (AUPR) for each sample. Next, we aggregated each individual sample’s performance by constructing bootstrapped confidence intervals. Ensuing model evaluations, we quantified the number of edges we could incorporate into Hetionet v1. We used our best-performing discriminative model to score every candidate sentence within our dataset and grouped candidates based on their mention pair. We took the max score within each candidate group, and this score represents the probability of the existence of an edge. We established edges using a cutoff score that produced an equal error rate between the false positives and false negatives. Lastly, we report the number of preexisting edges we could recall and the number of novel edges we can incorporate.

## Results

### Generative model using randomly sampled label functions

**Fig. 2:**
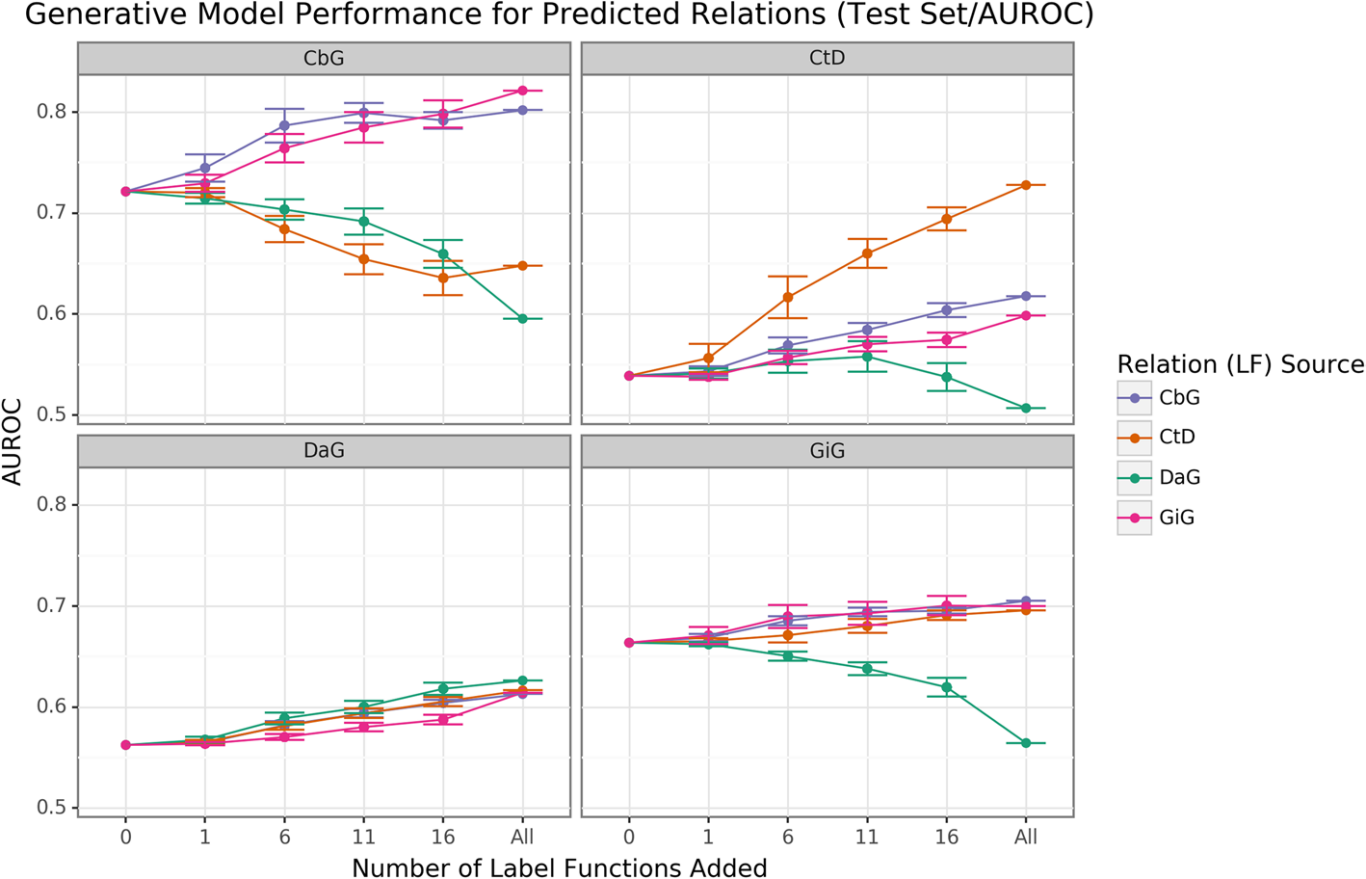
Edge-specific label functions perform better than edge-mismatch label functions, but certain mismatch situations show signs of successful transfer. Each line plot header depicts the edge type the generative model is trying to predict, while the colors represent the source of label functions. For example, orange represents sampling label functions designed to predict the Compound-treats-Disease (CtD) edge type. The x-axis shows the number of randomly sampled label functions incorporated as an addition to the database-only baseline model (the point at 0). The y-axis shows the area under the receiver operating curve (AUROC). Each point on the plot shows the average of 50 sample runs, while the error bars show the 95% confidence intervals of all runs. The baseline and “All” data points consist of sampling from the entire fixed set of label functions

Creating label functions is a labor-intensive process that can take days to accomplish. We sought to accelerate this process by measuring how well label functions can be reused. We evaluated this by performing an experiment where label functions are sampled on an individual (edge vs. edge) level and a global (collective pool of sources) level. We observed that performance increased when edge-specific label functions were added to an edge-specific baseline model, while label function reuse usually provided less benefit (AUROC Fig. 2, AUPR Supplemental Fig. 6). The quintessential example of this overarching trend is the Compound-treats-Disease (CtD) edge type, where edge-specific label functions consistently outperformed transferred label functions. However, there is evidence that label function transferability may be feasible for selected edge types and label function sources. Performance increases as more Gene-interacts-Gene (GiG) label functions are incorporated into the Compound-binds-Gene (CbG) baseline model and vice versa. This trend suggests that sentences for GiG and CbG may share similar linguistic features or terminology that allows for label functions to be reused, which could relate to both describing physical interaction relationships. Perplexingly, edge-specific Disease-associates-Gene (DaG) label functions did not improve performance over label functions drawn from other edge types. Overall, only CbG and GiG showed significant signs of reusability. This pattern suggests that label function transferability may be possible for these two edge types.

**Fig. 3:**
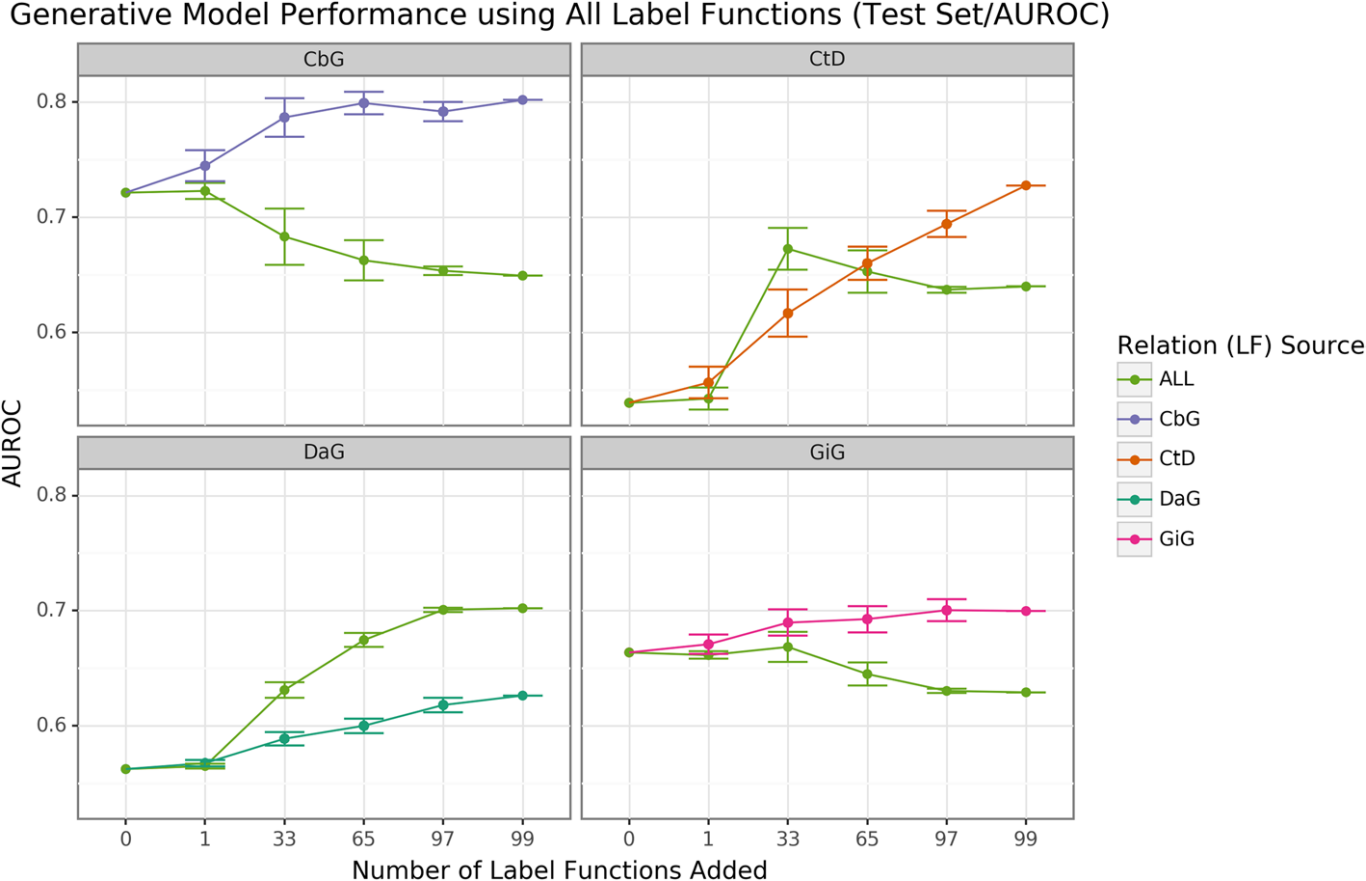
Using all label functions generally hinders generative model performance. Each line plot header depicts the edge type the generative model is trying to predict, while the colors represent the source of label functions. For example, orange represents sampling label functions designed to predict the Compound-treats-Disease (CtD) edge type. The x-axis shows the number of randomly sampled label functions incorporated as an addition to the database-only baseline model (the point at 0). The y-axis shows the area under the receiver operating curve (AUROC). Each point on the plot shows the average of 50 sample runs, while the error bars show the 95% confidence intervals of all runs. The baseline and “All” data points consist of sampling from the entire fixed set of label functions

We found that sampling from all label function sources at once usually underperformed relative to edge-specific label functions (Fig. 3 and Supplemental Fig. 7). The gap between edge-specific sources and all sources widened as we sampled more label functions. CbG is a prime example of this trend (Fig. 3 and Supplemental Fig. 7), while CtD and GiG show a similar but milder trend. DaG was the exception to the general rule. The pooled set of label functions improved performance over the edge-specific ones, which aligns with the previously observed results for individual edge types (Fig. 2). When pooling all label functions, the decreasing trend supports the notion that label functions cannot simply transfer between edge types (exception being CbG on GiG and vice versa).

### Discriminative model performance

**Fig. 4:**
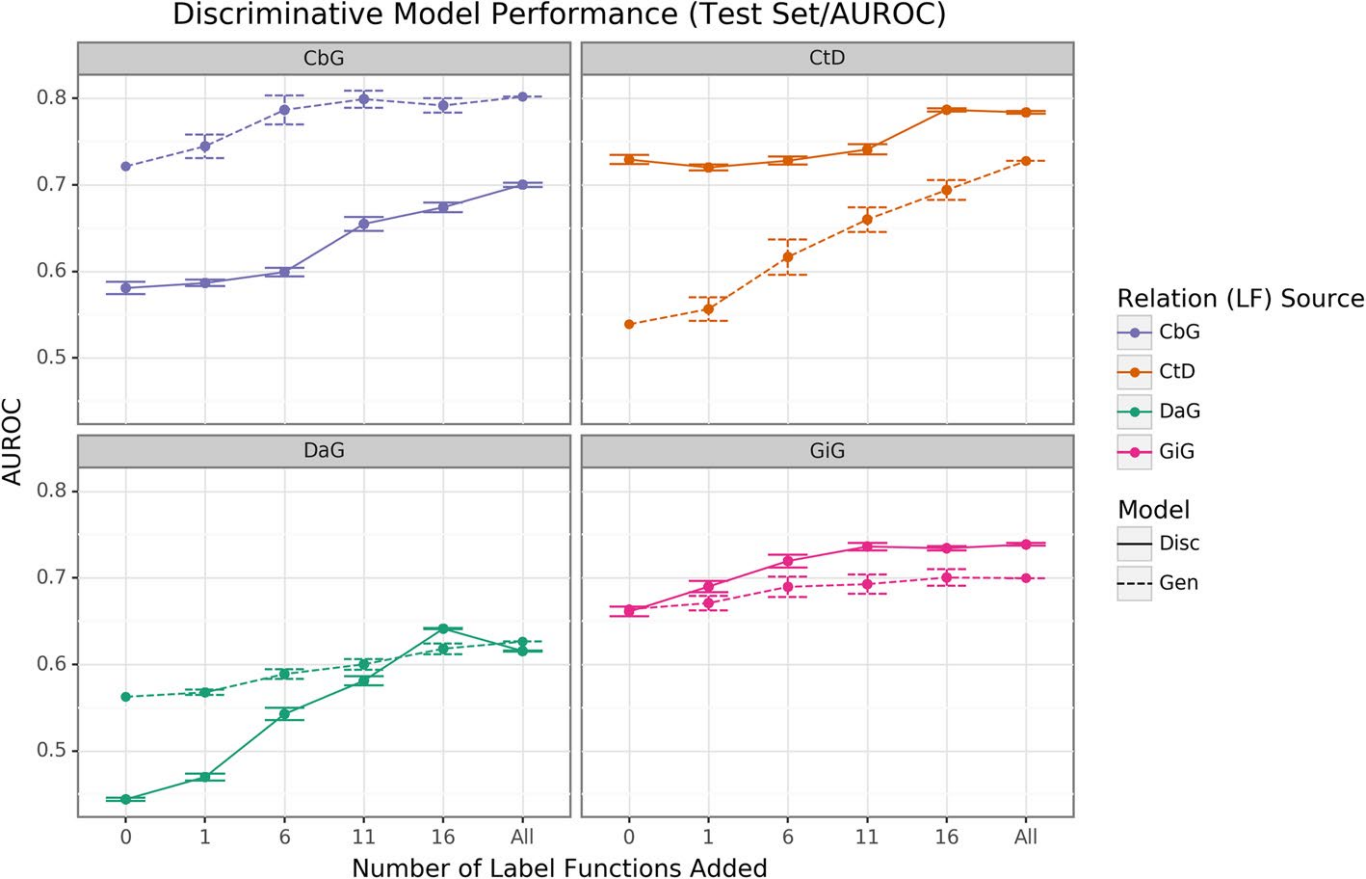
The discriminative model usually improves faster than the generative model as more edge-specific label functions are included. The line plot headers represent the specific edge type the discriminative model is trying to predict. The x-axis shows the number of randomly sampled label functions incorporated as an addition to the baseline model (the point at 0). The y axis shows the area under the receiver operating curve (AUROC). Each data point represents the average of 3 sample runs for the discriminator model and 50 sample runs for the generative model. The error bars represent each run’s 95% confidence interval. The baseline and “All” data points consist of sampling from the entire fixed set of label functions

The discriminative model is intended to augment performance over the generative model by incorporating textual features together with estimated training labels. We found that the discriminative model generally outperformed the generative model with respect to AUROC as more edge-specific label functions were incorporated (Fig. 4). Regarding AUPR, this model outperformed the generative model for the DaG edge type. At the same time, it had close to par performance for the rest of the edge types (Supplemental Fig. 8). The discriminative model’s performance was often poorest when very few edge-specific label functions were incorporated into the baseline model (seen in DaG, CbG, and GiG). This example suggests that training generative models with more label functions produces better outputs for training for discriminative models. CtD was an exception to this trend, where the discriminative model outperformed the generative model at all sampling levels in regards to AUROC. We observed the opposite trend with the CbG edges as the discriminative model was always worse or indistinguishable from the generative model. Interestingly, the AUPR for CbG plateaus below the generative model and decreases when all edge-specific label functions are used (Supplemental Fig. 8). This trend suggests that the discriminative model might have predicted more false positives in this setting. Overall, incorporating more edge-specific label functions usually improved performance for the discriminative model over the generative model.

### Text mined edges can expand a database-derived knowledge graph

**Fig. 5:**
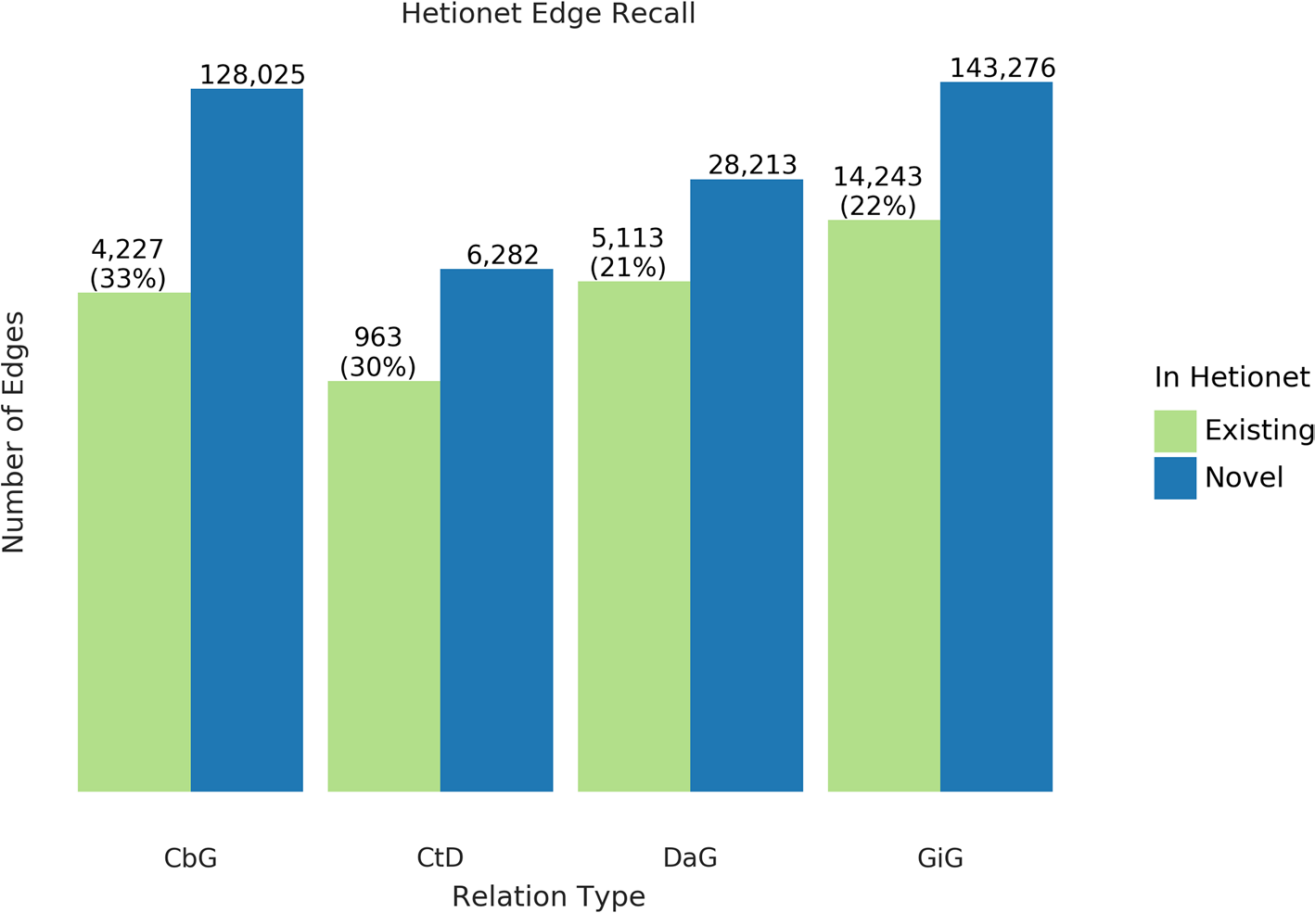
Text-mined edges recreate a substantial fraction of an existing knowledge graph and include new predictions. This bar chart shows the number of edges we can successfully recall in green and indicates the number of new edges in blue. The recall for the Hetionet v1 knowledge graph is shown as a percentage in parentheses. For example, for the Compound-treats-Disease (CtD) edge, our method recalls 30% of existing edges and can add 6,282 new ones

One of the goals of our work is to measure the extent to which learning multiple edge types could construct a biomedical knowledge graph. Using Hetionet v1 as an evaluation set, we measured this framework’s recall and quantified the number of edges that may be incorporated with high confidence. Overall, we were able to recall about thirty percent of the preexisting edges for all edge types (Fig. 5) and report our top ten scoring sentences for each edge type in Supplemental Table 3. Our best recall was with the CbG edge type, where we retained 33% of preexisting edges. In contrast, we only recalled close to 30% for CtD, while the other two categories achieved a recall score close to 22%. Despite the modest recall level, the amount of novel edge types remains elevated. This notion highlights that Hetionet v1 is missing a compelling amount of biomedical information, and relationship extraction is a viable way to close the information gap.

## Discussion

Filling out knowledge bases via manual curation can be an arduous and erroneous task [8]. Using manual curation alone becomes impractical as the rate of publications continuously increases. Data programming is a paradigm that uses label functions to speed up the annotation process and can be used to solve this problem. However, creating useful label functions is an obstacle to this paradigm, which takes considerable time. We tested the feasibility of re-using label functions to reduce the number of label functions required for strong prediction performance.

Our sampling experiment revealed that adding edge-specific label functions is better than adding off-edge label functions. An exception to this trend is using label functions designed from conceptually related edge types (using GiG label functions to predict CbG sentences and vice versa). Furthermore, broad edge types such as DaG did not follow this trend as we found this edge to be agnostic to any tested label function source. One possibility for this observation is that the “associates” relationship is a general concept that may include other concepts such as Disease (up/down) regulating a Gene (examples highlighted in our annotated sentences). These two results suggest that the transferability of label functions is likely to relate to the nature of the edge type in question, so determining how many label functions will be required to scale across multiple relationship types will depend on how conceptually similar those types are.

The discriminator model did not have an apparent positive or negative effect on performance; however, we noticed that performance heavily depended on the annotations provided by the generative model. This pattern suggests a focus on label function construction and generative model training may be key steps to focus on in future work. Although we found that label functions cannot be re-used across all edge types with the standard task framing, strategies like multitask [61] or transfer learning [62] may make multi-label-function efforts more successful.

## Conclusions

We found that performance often increased through the tested range of 25–30 different label functions per relationship type. Our finding of limited value for reuse across most edge type pairs suggests that the amount of work required to construct graphs will scale linearly based on the number of edge types. We did not investigate whether certain individual label functions, as opposed to the full set of label functions for an edge type, were particularly reusable. It remains possible that some functions are generic and could be used as the base through supplementation with additional, type-specific, functions. Literature continues to grow at a rate likely to surpass what is feasible by human curation. Further work is needed to understand how to automatically extract large-scale knowledge graphs from the wealth of biomedical text.

## Supplementary Information

Additional file 1.

Additional file 2.
