# Supplementary material for: Expanding a database-derived biomedical knowledge graph via multi-relation extraction from biomedical abstracts
Source: BioData Min. 2022 Oct 18;15:26. doi: 10.1186/s13040-022-00311-z (PMC9578183; doi:10.1186/s13040-022-00311-z)
Supplement: Supplementary file 1 — Additional file 1. [file 13040_2022_311_MOESM1_ESM.docx]

## Supplemental Figures

### Generative Model Using Randomly Sampled Label Functions

#### Individual Sources


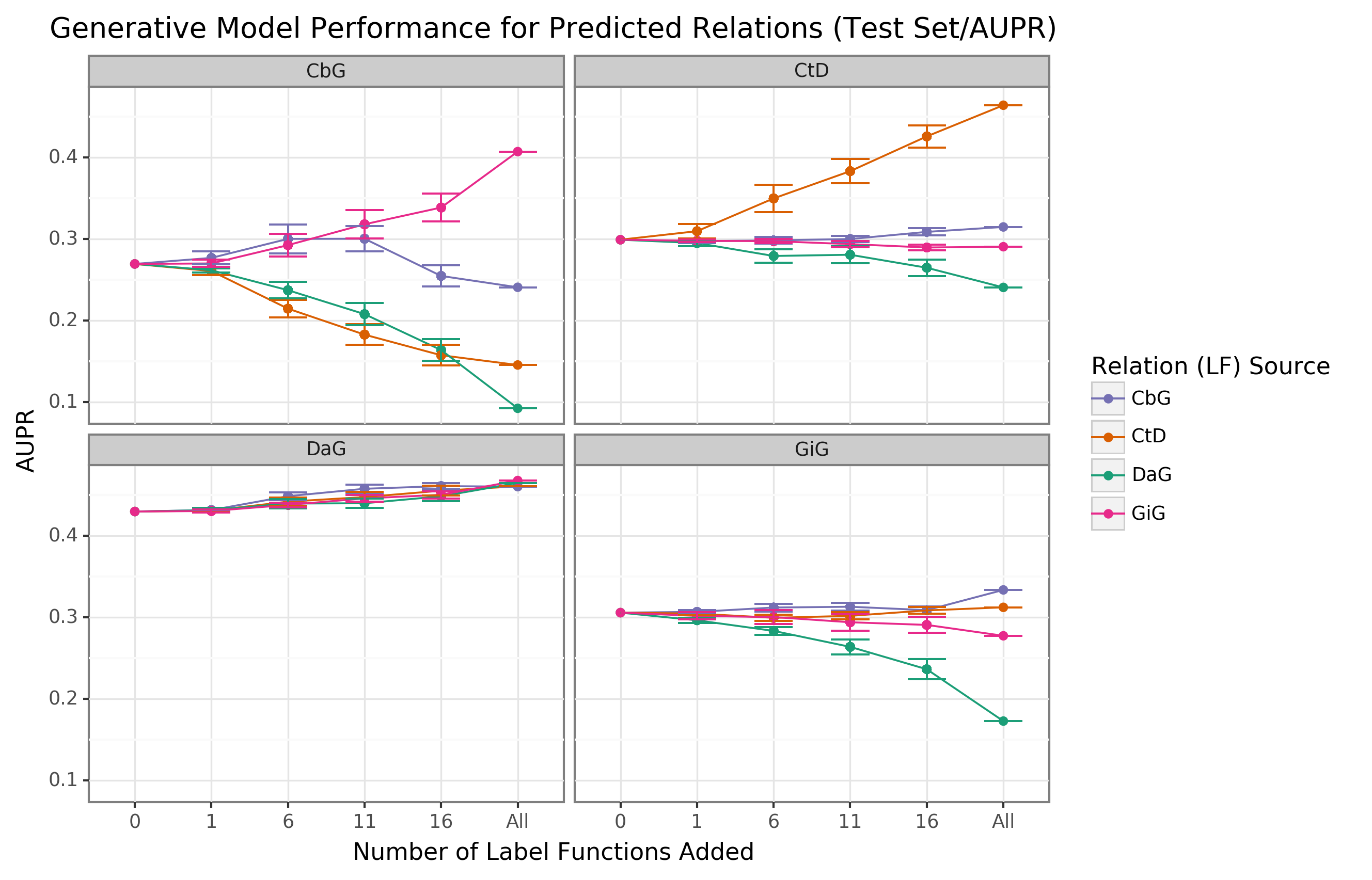


Figure 6: Edge-specific label functions improve performance over edge-mismatch label functions. Each line plot header depicts the edge type the generative model is trying to predict, while the colors represent the source of label functions. For example, orange represents sampling label functions designed to predict the Compound treats Disease (CtD) edge type. The x-axis shows the number of randomly sampled label functions incorporated as an addition to the database-only baseline model (the point at 0). The y-axis shows the area under the precision-recall curve (AUPR). Each point on the plot shows the average of 50 sample runs, while the error bars show the 95% confidence intervals of all runs. The baseline and “All” data points consist of sampling from the entire fixed set of label functions.

#### Collective Pool of Sources


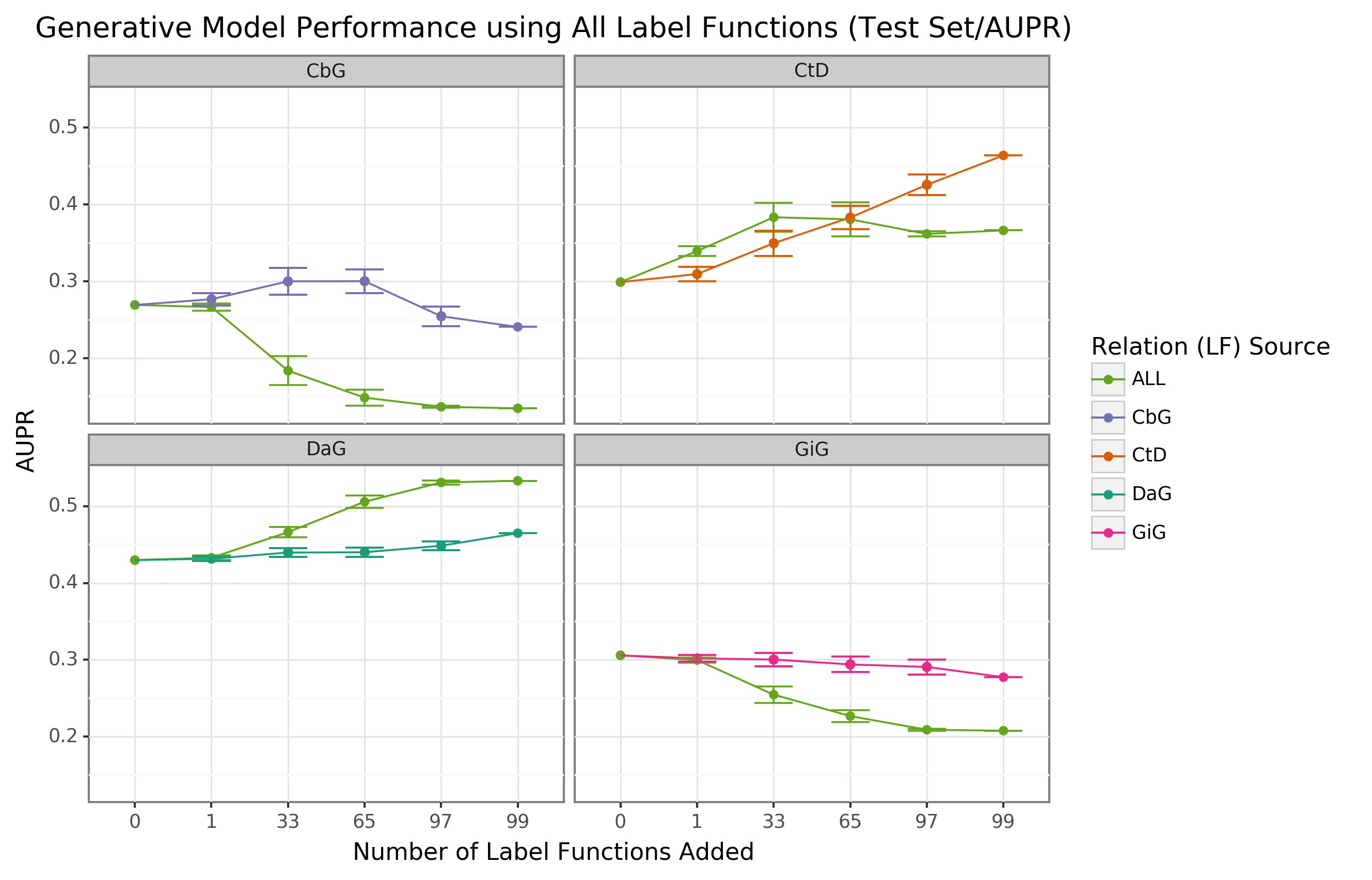


Figure 7: Using all label functions generally hinders generative model performance. Each line plot header depicts the edge type the generative model is trying to predict, while the colors represent the source of label functions. For example, orange represents sampling label functions designed to predict the Compound treats Disease (CtD) edge type. The x-axis shows the number of randomly sampled label functions incorporated as an addition to the database-only baseline model (the point at 0). The y-axis shows the area under the precision-recall curve (AUPR). Each point on the plot shows the average of 50 sample runs, while the error bars show the 95% confidence intervals of all runs. The baseline and “All” data points consist of sampling from the entire fixed set of label functions.

### Discriminative Model Performance


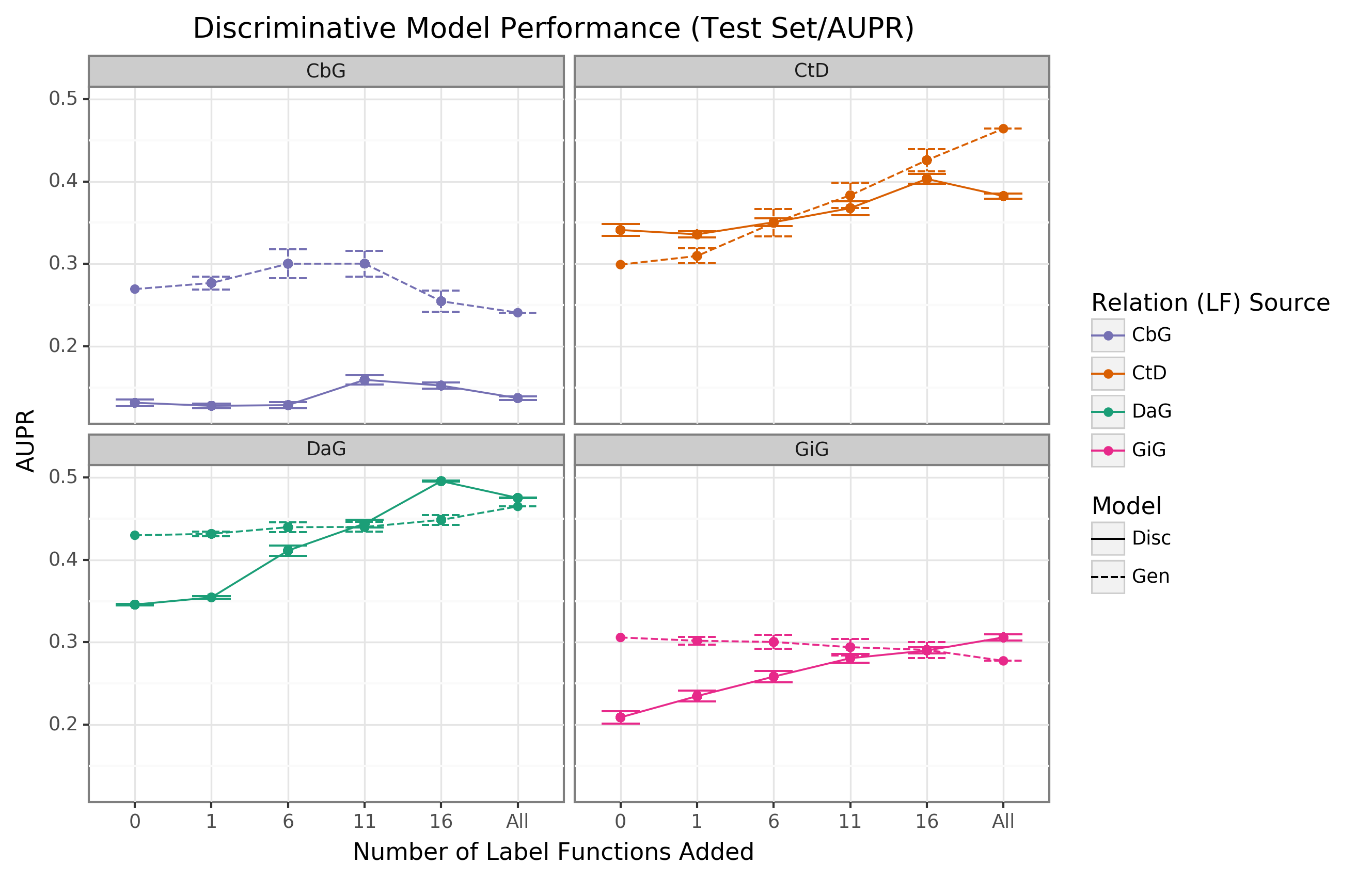


Figure 8: The discriminator model improves performance as the number of edge-specific label functions is added to the baseline model. The line plot headers represent the specific edge type the discriminator model is trying to predict. The x-axis shows the number of randomly sampled label functions incorporated as an addition to the baseline model (the point at 0). The y axis shows the area under the precision-recall curve (AUPR). Each data point represents the average of 3 sample runs for the discriminator model and 50 sample runs for the generative model. The error bars represent each run’s 95% confidence interval. The baseline and “All” data points consist of sampling from the entire fixed set of label functions.

## Supplemental Tables

### Top Ten Sentences for Each Edge Type

Table 3: Contains the top ten predictions for each edge type. Highlighted words represent entities mentioned within the given sentence.

| Edge Type | Source Node | Target Node | Generative Model Prediction | Discriminative Model Prediction | Number of Sentences | In Hetionet | Text |
| --- | --- | --- | --- | --- | --- | --- | --- |
| DaG | hematologic cancer | STMN1 | 1.000 | 0.979 | 83 | Novel | the stathmin1 mrna expression level in de novo al patient be high than that in healthy person ( p < 0.05 ) , the [stathmin1].{gene_color} mrna expression level in relapse patient with al be high than that in de novo patient ( p < 0.05 ) , and there be no significant difference of stathmin1 mrna expression between patient with [aml].{disease_color} and patient with all . |
| DaG | breast cancer | INSIG2 | 1.000 | 0.979 | 4 | Novel | in analysis of [idc ].{disease_color} cell , the level of [insig2].{gene_color} mrna expression be significantly high in late - stage patient than in early - stage patient . |
| DaG | lung cancer | GNAO1 | 1.000 | 0.979 | 104 | Novel | high [numb].{disease_color} expression be associate with favorable prognosis in patient with [lung adenocarcinoma].{gene_color} , but not in those with squamous cell carcinoma . |
| DaG | breast cancer | TTF1 | 1.000 | 0.977 | 88 | Novel | significant [ttf-1].{gene_color} overexpression be observe in adenocarcinomas harbor egfr mutation ( p = 0.008 ) , and no or significantly low level expression of ttf-1 be observe in [adenocarcinomas].{disease_color} harbor kras mutation ( p = 0.000 ) . |
| DaG | breast cancer | BUB1B | 1.000 | 0.977 | 13 | Novel | elevated [bubr1].{gene_color} expression be associate with poor survival in early stage [breast cancer].{disease_color} patient . |
| DaG | Alzheimer’s disease | SERPINA3 | 1.000 | 0.977 | 182 | Existing | a common polymorphism within act and il-1beta gene affect plasma level of [act].{gene_color} or il-1beta , and [ad].{disease_color} patient with the act t , t or il-1beta t , t genotype show the high level of plasma act or il-1beta , respectively . |
| DaG | esophageal cancer | TRAF6 | 1.000 | 0.976 | 15 | Novel | expression of traf6 be highly elevated in [esophageal cancer].{disease_color} tissue , and patient with high [traf6].{gene_color} expression have a significantly short survival time than those with low traf6 expression . |
| DaG | hypertension | TBX4 | 1.000 | 0.975 | 146 | Novel | the proportion of circulate [th1].{gene_color} cell and the level of t - bet , ifng mrna be increase in [ht].{disease_color} patient , the expression of ifng - as1 be upregulated and positively correlate with the proportion of circulate th1 cell or t - bet , and ifng expression , or serum level of anti - thyroglobulin antibody / thyroperoxidase antibody in ht patient . |
| DaG | breast cancer | TP53 | 1.000 | 0.975 | 3481 | Existing | hormone receptor status rather than her2 status be significantly associate with increase ki-67 and [p53].{gene_color} expression in triple [- negative ].{disease_color} breast carcinoma , and high expression of ki-67 but not p53 be significantly associate with axillary nodal metastasis in triple - negative and high - grade non - triple - negative breast carcinoma . |
| DaG | esophageal cancer | COL17A1 | 1.000 | 0.975 | 32 | Novel | high [cd147].{gene_color} expression in patient with [esophageal cancer].{disease_color} be associate with bad survival outcome and common clinicopathological indicator of poor prognosis . |
| CtD | Docetaxel | prostate cancer | 0.996 | 0.964 | 5614 | Existing | docetaxel and atrasentan versus [docetaxel ].{compound_color} and placebo for man with advanced castration - resistant [prostate cancer].{disease_color} ( swog s0421 ) : a randomised phase 3 trial |
| CtD | E7389 | breast cancer | 0.999 | 0.957 | 862 | Novel | clinical effect of prior trastuzumab on combination [eribulin mesylate].{compound_color} plus trastuzumab as first - line treatment for human epidermal growth factor receptor 2 positive locally recurrent or metastatic [breast cancer].{disease_color} : result from a phase ii , single - arm , multicenter study |
| CtD | Zoledronate | bone cancer | 0.996 | 0.955 | 226 | Novel | [zoledronate].{compound_color} in combination with chemotherapy and surgery to treat [osteosarcoma].{disease_color} ( os2006 ) : a randomised , multicentre , open - label , phase 3 trial . |
| CtD |  |  | 0.878 | 0.954 | 484 | Existing | the role of [ixazomib].{compound_color} as an augment conditioning therapy in salvage autologous stem cell transplant ( asct ) and as a post - asct consolidation and maintenance strategy in patient with relapse multiple myeloma ( accord [ uk - mra [myeloma].{disease_color} xii ] trial ) : study protocol for a phase iii randomise controlled trial |
| CtD | Topotecan | lung cancer | 1.000 | 0.954 | 315 | Existing | combine chemotherapy with cisplatin , etoposide , and irinotecan versus [topotecan].{compound_color} alone as second - line treatment for patient with [sensitive relapse small].{disease_color} - cell lung cancer ( jcog0605 ) : a multicentre , open - label , randomised phase 3 trial . |
| CtD | Epirubicin | breast cancer | 0.999 | 0.953 | 2147 | Existing | accelerate versus standard [epirubicin].{compound_color} follow by cyclophosphamide , methotrexate , and fluorouracil or capecitabine as adjuvant therapy for [breast cancer].{disease_color} in the randomised uk tact2 trial ( cruk/05/19 ) : a multicentre , phase 3 , open - label , randomise , control trial |
| CtD | Paclitaxel | breast cancer | 1.000 | 0.952 | 10255 | Existing | sunitinib plus [paclitaxel].{compound_color} versus bevacizumab plus paclitaxel for first - line treatment of patients with [advanced breast cancer].{disease_color} : a phase iii , randomized , open - label trial |
| CtD | Anastrozole | breast cancer | 0.996 | 0.952 | 2364 | Existing | a european organisation for research and treatment of cancer randomize , double - blind , placebo - control , multicentre [phase].{disease_color} ii trial of anastrozole in combination with [gefitinib or placebo in hormone].{compound_color} receptor - positive advanced breast cancer ( nct00066378 ) . |
| CtD | Gefitinib | lung cancer | 1.000 | 0.950 | 11860 | Existing | [gefitinib].{compound_color} versus placebo as maintenance therapy in patient with locally advanced or metastatic [non - small].{disease_color} - cell lung cancer ( inform ; c - tong 0804 ) : a multicentre , double - blind randomise phase 3 trial . |
| CtD | Docetaxel | prostate cancer | 1.000 | 0.949 | 5614 | Existing | ipilimumab versus placebo after radiotherapy in patient with metastatic castration - resistant [prostate cancer].{disease_color} that have progress after [docetaxel].{compound_color} chemotherapy ( ca184 - 043 ) : a multicentre , randomised , double - blind , phase 3 trial |
| CtD | Sulfamethazine | lung cancer | 0.611 | 0.949 | 4 | Novel | [tmp].{compound_color} / smz ( 320/1600 mg / day ) treatment be compare to placebo in a double - blind , randomized trial in [patient with newly diagnose].{disease_color} small cell carcinoma of the lung during the initial course of chemotherapy with cyclophosphamide , doxorubicin , and etoposide . |
| CbG | D-Tyrosine | EGFR | 0.601 | 0.876 | 3423 | Novel | amphiregulin ( ar ) and heparin - binding egf - like growth factor ( hb - [egf].{gene_color} ) bind and activate the egfr while heregulin ( hrg [) act ].{compound_color} through the p185erbb-2 and p180erbb-4 tyrosine kinase . |
| CbG | Phosphonotyrosine | ANK3 | 0.004 | 0.865 | 1 | Novel | at least two domain of p85 can bind to [ank3 ].{gene_color} , and the interaction involve the p85 c - sh2 domain be find to be [phosphotyrosine].{compound_color} - independent . |
| CbG | Adenosine | ABCC8 | 0.891 | 0.860 | 353 | Novel | sulfonylurea act by inhibition of [beta - cell ].{compound_color} adenosine triphosphate - dependent potassium ( k(atp ) ) channel after bind to the sulfonylurea subunit 1 [receptor ( ].{gene_color} sur1 ) . |
| CbG | D-Tyrosine | AREG | 0.891 | 0.857 | 22 | Novel | amphiregulin ( [ar ) ].{gene_color} and heparin - binding egf - like growth factor ( hb - egf ) bind and activate the egfr while heregulin ( hrg [) act ].{compound_color} through the p185erbb-2 and p180erbb-4 tyrosine kinase . |
| CbG | D-Tyrosine | EGF | 0.602 | 0.856 | 389 | Novel | upon activation of the receptor for the epidermal growth factor ( [egfr ) ].{gene_color} , sprouty2 undergoe phosphorylation at a conserve [tyrosine ].{compound_color} that recruit the src homology 2 domain of c - cbl . |
| CbG | D-Tyrosine | CSF1 | 0.101 | 0.854 | 106 | Novel | as a member of the subclass iii family of receptor [tyrosine].{compound_color} kinase , kit be closely relate to the receptor for platelet derive growth factor alpha and beta ( pdgf - a and b [) , macrophage colony ].{gene_color} stimulate factor ( m - csf ) , and flt3 ligand . |
| CbG | D-Tyrosine | ERBB4 | 0.101 | 0.848 | 115 | Novel | the efgr family be a group of four structurally similar [tyrosine ].{compound_color} kinase ( egfr , her2 / neu , erbb-3 [, and erbb-4].{gene_color} ) that dimerize on bind with a number of ligand , include egf and transform growth factor alpha . |
| CbG | D-Tyrosine | EGFR | 0.969 | 0.848 | 3423 | Novel | the [epidermal growth factor receptor ].{gene_color} be a member of type - -pron- growth factor receptor [family ].{compound_color} with tyrosine kinase activity that be activate follow the binding of multiple cognate ligand . |
| CbG | D-Tyrosine | VAV1 | 0.601 | 0.842 | 187 | Novel | stimulation of quiescent rodent fibroblast with either epidermal or platelet - derive growth factor induce an increase affinity of vav for cbl - b and result in the [subsequent ].{gene_color} formation of a vav - [dependent ].{compound_color} trimeric complex with the ligand - stimulate tyrosine kinase receptor . |
| CbG | Tretinoin | RORB | 0.601 | 0.840 | 7 | Novel | the retinoid z receptor beta ( [rzr beta ) ].{gene_color} , an orphan receptor , be a member of the [retinoic acid].{compound_color} receptor ( rar)/thyroid hormone receptor ( tr ) subfamily of nuclear receptor . |
| CbG | L-Tryptophan | TACR1 | 0.891 | 0.839 | 4 | Novel | these result suggest that the [tryptophan ].{compound_color} and quinuclidine series of nk-1 antagonist bind to similar bind site on the human [nk-1 receptor ].{gene_color} . |
| GiG | CYSLTR2 | CYSLTR2 | 0.967 | 0.564 | 37 | Novel | the bind pocket of [cyslt2 ].{gene2_color} receptor and the proposition of the interaction mode between [cyslt2 ].{gene1_color} and hami3379 be identify . |
| GiG | RXRA | PPARA | 1.000 | 0.563 | 143 | Novel | after bind ligand , the [ppar ].{gene2_color} - y receptor heterodimerize [with ].{gene1_color} the rxr receptor . |
| GiG | RXRA | RXRA | 0.824 | 0.551 | 1101 | Existing | nuclear hormone receptor , for example , bind either as homodimer or as heterodimer with [retinoid x receptor ].{gene1_color} ( [rxr ) ].{gene2_color} to half - site repeat that be stabilize by protein - protein interaction mediate by residue within both the dna- and ligand - bind domain . |
| GiG | ADRBK1 | ADRA2A | 0.822 | 0.543 | 3 | Novel | mutation of these residue within the [holo - alpha(2a)ar diminish grk2-promoted].{gene2_color} phosphorylation [of ].{gene1_color} the receptor as well as the ability of the kinase to be activate by receptor binding . |
| GiG | ESRRA | ESRRA | 0.001 | 0.531 | 308 | Existing | the crystal structure of the ligand bind domain ( lbd ) of the estrogen - relate receptor [alpha ].{gene2_color} ( [erralpha , ].{gene1_color} nr3b1 ) complexe with a coactivator peptide from peroxisome proliferator - activate receptor coactivator-1alpha ( pgc-1alpha ) reveal a transcriptionally active conformation in the absence of a ligand . |
| GiG | GP1BA | VWF | 0.518 | 0.527 | 144 | Existing | these finding indicate the novel bind site require for [vwf ].{gene2_color} binding of human [gpibalpha ].{gene1_color} . |
| GiG | NR2C1 | NR2C1 | 0.027 | 0.522 | 26 | Novel | the human [testicular receptor 2].{gene1_color} ( [tr2 )].{gene2_color} , a member of the nuclear hormone receptor superfamily , have no identify ligand yet . |
| GiG | NCOA1 | ESRRG | 0.992 | 0.518 | 1 | Novel | the crystal structure of the ligand bind domain ( lbd ) of the estrogen - relate receptor [3 (].{gene2_color} err3 ) complexe with a steroid receptor [coactivator-1 (].{gene1_color} src-1 ) peptide reveal a transcriptionally active conformation in absence of any ligand . |
| GiG | PPARG | PPARG | 0.824 | 0.504 | 2497 | Existing | although these agent can bind and activate an orphan nuclear receptor , [peroxisome proliferator - activate].{gene2_color} receptor [gamma ( ].{gene1_color} ppargamma ) , there be no direct evidence to conclusively implicate this receptor in the regulation of mammalian glucose homeostasis . |
| GiG | ESR2 | ESR1 | 0.995 | 0.503 | 1715 | Novel | ligand bind experiment with purify [er alpha].{gene2_color} and [er beta].{gene1_color} confirm that the two phytoestrogen be er ligand . |
| GiG | FGFR2 | FGFR2 | 1.000 | 0.501 | 584 | Existing | receptor modeling of [kgfr].{gene1_color} be use to identify selective kgfr tyrosine kinase ( tk ) inhibitor molecule that have the potential to bind selectively to the [kgfr].{gene2_color} . |
